# fMRI exploration of pedagogical benefits of repeated testing: when more is not always better

**DOI:** 10.1002/brb3.476

**Published:** 2016-05-18

**Authors:** Xiaonan L. Liu, Lynne M. Reder

**Affiliations:** ^1^ Institute of PsychologySchool of Public PolicyXiamen UniversityXiamen361005China; ^2^ Department of PsychologyCarnegie Mellon UniversityPittsburghPennsylvania15213; ^3^ Center for the Neural Basis of CognitionCarnegie Mellon UniversityPittsburghPennsylvania15213

**Keywords:** Encoding, fMRI, retrieval, subsequent memory effect, testing effect

## Abstract

**Introduction:**

The testing effect refers to superior retention when study is followed by a test rather than followed by another study. Most research to date on why the testing effect occurs has been behavioral, but we employed neuroimaging methods in this study in order to shed light on the underlying processes.

**Methods:**

Subjects were scanned while studying, restudying, and taking cued‐recall tests of word pairs (with no feedback). We analyzed the BOLD responses by back sorting the encoding and test trials based on whether the subsequent test was correct or incorrect. We compared the subsequent memory patterns in initial study, restudy, and test trials.

**Results:**

Overall, brain activity during test trials was a better predictor of later performance than brain activity during restudy trials. For test trials, we separately examined brain regions associated with the retrieval attempt process during successful retrieval and regions associated with the re‐encoding process during retrieval in terms of prediction of subsequent memory. Regions associated with retrieval attempts were found to always predict subsequent memory success (the greater the activation, the more likely the correct recall); however, the regions associated with re‐encoding would sometimes predict subsequent failure, specifically when subjects had correctly recalled the associated word several times already.

**Conclusions:**

These results suggest that whether a testing effect advantage is observed depends on both on the retrieval process and the re‐encoding process which follows that retrieval.

## Introduction

Considerable research has demonstrated the benefits of memory retrieval on subsequent memory, highlighting a phenomenon known as the testing effect (Carrier and Pashler 1992; Roediger and Karpicke 2006a,b; McDaniel et al. 2007; Pashler et al. 2007; Karpicke and Roediger 2008; Rowland 2014). A prototypical experiment that demonstrates the facilitative effect of testing (e.g., Karpicke and Roediger 2008) involves subjects learning paired‐associative items by initially studying the pairs and after having subsequent learning trials for each of the pairs. Some pairs are restudied and others are tested. Memory for all pairs assessed on a final test typically shows superior performance for pairs that had an intervening test compared with pairs that had received additional study opportunities.

Contemporary theoretical explanations of the testing effect tend to focus on one or the other of the two processes that we believe are involved. Some theories focus on a *retrieval attempt process* in which a search is initiated to find the answer to the question. Such theories emphasize the role of retrieval per se, meaning that the process of searching for an answer will provide additional or stronger retrieval paths that facilitate subsequent retrieval attempts at later tests. An example of this theory is the *elaborative retrieval account*. The elaborative retrieval account states that the retrieval of information from memory results in memory elaborations that provide new routes to access the information. These additional retrieval routes make future attempts to access the information more likely to be successful (Anderson and Reder 1979; Carpenter and DeLosh 2006; Carpenter 2009). Another example of this type of explanation is the *episodic context account* of Karpicke et al. (2014) that states that the process of retrieval serves to increase the specificity of the search process by adding unique contextual elements to the trace thereby making retrieval easier on subsequent tests.

Other theories tend to focus on a *re‐encoding process* postulated to occur after a successful retrieval attempt (i.e., when the correct answer is in working memory). An example of this theory is the *reconsolidation account* (Finn and Roediger 2011). *Reconsolidation* refers to the idea that when information is retrieved from memory, it enters a *labile state*, rendering it amenable to change (Dudai 2004). The claim is that after the first successful recall of the studied information, the retrieved information enters an unstable state, thereby enabling the memory trace to be strengthened by the postretrieval re‐encoding of the correctly retrieved information. Note that one need not postulate reconsolidation or a labile state in order to posit a strengthening of the memory trace by virtue of having been retrieved. The important difference between the two accounts of the testing effect is that the first focuses on the process of search (finding paths to retrieve the information), while the other focuses on the opportunity to re‐encode (i.e., strengthen) the desired information that was found.

While both types of accounts of the testing effect can explain some of the existing results (see Roediger and Butler 2011, for a review), there remain contradictory findings that neither class of theories can explain. For example, one assumption accepted by many in the field is that the benefit from one single test is limited and the effect of multiple tests is larger than would be expected as a simple multiplier of a single test. Such a pattern suggests that multiple tests are necessary to maximize the benefits of testing (e.g., Karpicke and Roediger 2008; Vaughn et al. 2013). On the other hand, other studies have shown that the value added of each additional test beyond the first provides little improvement over a single test prior to the final assessment (Roediger and Karpicke 2006b; Pyc and Rawson 2009).

Given these inconsistent patterns, we sought a theoretical explanation that could capture these different results within a single account. We speculated that the two theoretical accounts reviewed above might both contribute to the testing effect. Specifically, depending on the relative contribution of these two processes (retrieval and re‐encoding) in a particular paradigm, one would find a pattern consistent with the view that a single test suffices or that there is greater benefit with multiple tests.

In our view these two processes, retrieval attempt and re‐encoding, are dissociable because individuals might become less likely to re‐encode retrieved information when it has been answered correctly multiple times already. Typically, the first retrieval attempt of stored information during testing will (1) facilitate and strengthen the traces involved in that retrieval activity, and (2) the subject will also engage in a postretrieval re‐encoding process of the correct information, thereby also strengthening the target trace. The tendency to re‐encode, however, may become less likely with each additional successful retrieval. The notion that there exist two dissociable processes was suggested by our previous neuroimaging findings (Liu et al. 2014).

The evidence suggesting these different processes in the Liu et al. study were uncovered by employing a subsequent memory analysis on the test trials as opposed to the encoding trials. Previously, subsequent memory analyses had been used to back sort encoding trials based on whether the trial was successfully remembered later (Wagner et al. 1998). By focusing on the neural signatures of successful retrievals, Liu et al. found several patterns not previously documented: First, the brain regions in the left hemisphere, including prefrontal cortex (PFC), posterior parietal cortex (PPC), and hippocampus (HPC) that had previously been shown to predict later performance during encoding are also active during retrieval and also predictive of later performance. Second, there are other regions in the right hemisphere, specifically right PFC and right PPC, whose activation patterns are only predictive of subsequent performance during retrieval, not during encoding.

In addition, that experiment used feedback after each test so they were able to examine the effect of restudy after testing. They found that when feedback is provided after attempting to answer the test question, the restudy activation is only predictive of subsequent memory performance when the test answer that preceded feedback had been wrong. This result suggests if the subject is told that he or she is correct based on the feedback, the subject does not bother to “restudy” the paired associate. In the current experiment, we want to explore whether allocation of effort to the re‐encoding process during retrieval is also affected by ease of retrieval even when there is no feedback. In other words, we want to examine whether the activity of the re‐encoding process of successful retrieval is similar to the activity during restudy trials as observed in Liu et al. In that experiment, brain activity following feedback only predicted subsequent learning when the answer had been wrong. Here, the question is will re‐encoding activation following a (correct) retrieval attempt but with no feedback only predict subsequent learning when the retrieved answer is not well learned.

In this experiment, we use multiple tests during the learning phase in order to manipulate the degree of overlearning/ease of retrieval. The learning phase takes place in the scanner and the final assessment occurs a day later, outside the scanner. We employ a subsequent memory analysis and use the regions of interest (ROIs) identified from our previous fMRI study (Liu et al. 2014). In this study, we only examine brain activity associated with correct intervening tests in different conditions because without feedback and restudy opportunities that follow feedback, subjects will not learn from incorrect tests. We compare subsequent memory patterns between brain activity associated with the first and the second test. In this way we can tease apart the contributions of retrieval and re‐encoding neural processes and examine the contribution of these processes as a function of learning conditions.

## Materials and Methods

### Subjects

Twenty subjects (nine females, mean age 20 ± 1.22), all attending Carnegie Mellon University, with normal or corrected‐to‐normal vision participated in two sessions with 1 day between them. They were paid after completion of both sessions. One subject was excluded from the fMRI analyses due to excessive head motion. This study was approved by Carnegie Mellon University Institutional Review Board (IRB) and all subjects were treated in accordance with the CMU IRB guidelines.

### Design

**Figure 1:**
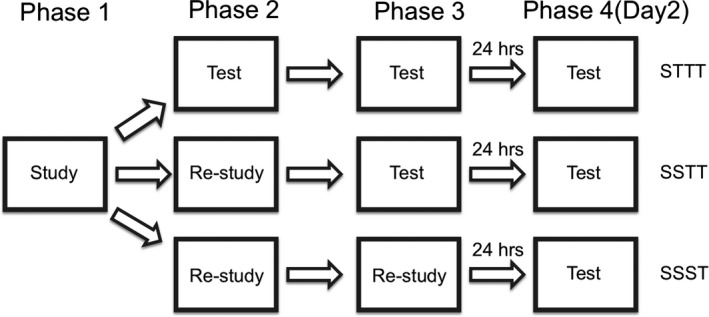
Illustration of the three treatment conditions for word pairs on a given list. Phases 1, 2, and 3 occurred in the scanner on Day 1. Phase 4 was administered outside of the scanner on Day 2.

A within‐subject design was employed with three levels of a single factor, type of training for learning arbitrary word paired associates. There were three dependent measures: accuracy on cued recall of the response term of the word pair; reaction time (RT) to give the correct answer, and BOLD response on restudy and test trials. Figure 1 illustrates the three training conditions for individual word pairs. All conditions involved an initial encoding (study) trial denoted as S. All conditions ended with a final test (‐T) in a separate session outside the scanner. The top row illustrates the STT‐T condition denoting that pairs in this condition were tested twice after initial encoding before the final test on the second day. The next row of Figure 1 shows that pairs in the SST‐T condition get one restudy opportunity after initial encoding followed by one test before the final test on Day 2. The bottom row of Figure 1 illustrates that pairs in the SSS‐T condition only had study opportunities on Day 1 and were only tested on the second day on the final test.

### Materials

We selected 320 words from the MRC database (Wilson 1988) with the following constraints: 4–7 letters in length and ratings between 500 and 700 for printed familiarity, concreteness, and imageability. For each subject, 272 words were randomly selected from the pool of 320 (without replacement) and paired with other words and assigned to the three different types of learning conditions (also done randomly for each subject). Word pairs were also randomly assigned to four separate lists so that subjects only needed to study 34 pairs at a time. Pilot testing indicated that with longer lists, there were not enough correct responses in Phase 2. Each of the four lists consisted of 34 paired associates, 17 for the STT‐T condition, 11 for the SST‐T condition, and 6 for the SSS‐T condition. The rationale for an unequal number of trials for the different conditions was based on the need for sufficient observations of correct responses in the conditions of particular interest. Our analyses focus primarily on the subsequent memory effects from correct test trials. In order to insure sufficient numbers of correct trials in each test phase, we assigned more word pairs to conditions with more testing.

### Procedure

#### Procedural overview

Phases 1–3 occurred in the scanner while the final phase (4) is outside the scanner on Day 2. Phases 1 and 2 of learning occurred for each of the four lists before any of the lists transitioned to Phase 3 of learning.

As shown in Figure 1, for Phase 1, subjects studied all the pairs of List 1 once and then subjects were given the opportunity to better learn each pair once more in Phase 2. In the second phase, some pairs were presented for restudy while other pairs were tested. Testing involved presenting the left‐hand word of a pair and asking the subject to try to recall its corresponding right‐hand word. The details of how subjects could respond in the scanner are described below under the “Procedural details during scanning” section. There was no feedback after the recall attempt.

Before subjects continued the learning of pairs of the first list (in Phase 3), subjects began studying the next of the four lists. Following study of all those word pairs, those pairs received additional training in Phase 2 as described earlier. This was repeated for the remaining two lists (total four) before initiation of Phase 3.

After all four lists had gone through Phase 1 and Phase 2 training, and the four lists were recombined into four new lists for Phase 3. Whether a word pair was tested or restudied in Phase 3 depends on its initial assignment to condition.

#### Procedural details during scanning

The session on Day 1 lasted about 80 min in the scanner. For Phase 1, word pairs were presented one at a time to study for 3 sec. Each study trial began with a fixation cross for a jittered period of either 1 or 3 sec. In Phases 2 and 3, restudy trials were the same as Phase 1. Test trials in Phases 2 and 3 also began with a fixation cross for either 1 or 3 sec, followed by the cue word in the center of the screen with a question mark prompt to indicate that the subject should try to recall the response term.^1^ All tests were self‐paced with a time limit of 8 sec. To circumvent the problems of typing words while in the scanner subjects indicated the correct response term from a list of words shown on the sides of the screen, displayed in alphabetical order with a three‐digit number listed underneath each choice (see Liu et al. 2014, for more details). The 34 alternatives were shown simultaneously when the cue word was displayed. These words were all the response terms for pairs in that list (34 alternatives). Subjects were trained to key in three digit numbers, using a data glove before going into the scanner and then practiced this skill while structural images were taken. Subjects were instructed that the items would be displayed alphabetically and to first recall the answer and then locate that word on the screen. Subjects were also instructed to give their best guess when they could not recall an answer (there was no option to respond “Don't Know”). We discuss this procedure for responding in more detail in the Results and Discussion.

#### Details for Phase 4 on second day

The session on Day 2 lasted about 45 min and it was conducted in a behavioral laboratory. For Phase 4 (occurred on Day 2 outside the scanner), test trials began with a fixation cross for 2 sec, followed by the cue word in the center of the screen with a question mark prompt to indicate that the subject should try to recall and type the whole target word. Subjects were paid at the end of the session on Day 2.

### fMRI data acquisition

The fMRI experiment was conducted using a Siemens 3T Verio MRI system (Siemens Medical Solutions, Erlangen, Germany). A high‐resolution structural image (0.8 × 0.8 × 0.8 mm) was acquired using magnetization‐prepared rapid acquisition with gradient echo (MPRAGE) (time repetition [TR] = 1800 ms, time echo [TE] = 2.22 ms, field of view [FOV] = 205, flip angle [FA] = 9°, number of slices = 256).

Functional data were collected using a gradient‐echo, echo‐planar sequence (TR = 2000 ms, TE = 30 ms, FOV = 205, FA = 79°, 36 slices, 3.2 × 3.2 × 3.2 mm).

### fMRI data analysis

Data were analyzed using SPM8 software (RRID:nif‐0000‐00343; http://www.fil.ion.ucl.ac.uk). The fMRI images were first corrected for within‐scan acquisition time differences between slices and then realigned to the first volume to correct for interscan head motions. The structural image was coregistered to the mean functional image created from the realigned images using a linear transformation. The transformed structural images were then segmented into gray matter (GM), white matter (WM), and cerebrospinal fluid (CSF) by using a unified segmentation algorithm. The realigned functional volumes were spatially normalized to the Montreal Neurological Institute (MNI) space and resampled to 3 mm isotropic voxels using the normalization parameters estimated during unified segmentation. The registration of the functional data to the template was checked for each individual participant. Subsequently, the functional images were spatially smoothed with a Gaussian kernel of 6 × 6 × 6 mm^3^ full width at half maximum (FWHM) to decrease spatial noise. The BOLD signal was modeled using canonical Haemodynamic Response Function (HRF) with time derivative implemented in SPM8.

Six ROIs, bilateral PFC, bilateral PPC, and bilateral HPC, were included in the predefined analyses. All ROIs were functionally defined based on a meta‐analysis of subsequent memory effects of memory encoding studies involving both verbal and spatial materials and both recognition and recall tasks (Kim 2011) using the WFU Pick Atlas toolbox (Maldjian et al. 2003, 2004). In addition, these regions have also been associated with memory retrieval in other studies (Tulving et al. 1994; Buckner et al. 1995; Nolde et al. 1998). These ROIs were also used in our prior study that examined the subsequent memory effect based on activation during retrieval on the first test (Liu et al. 2014). The centroid MNI coordinates for each ROI were as follows: left PFC (−46 26 16), right PFC (48 6 30), left PPC (−28 −76 36), right PPC (26 −62 46), left HPC (−22 −10 −16), and right HPC (18 −8 −16). All ROIs were defined as cubes of 9 × 9 × 9 mm^3^. For test trials, the epoch of interest was from the presentation of the cue word until the response. The MarsBaR toolbox (Brett et al. 2002) was used to extract the beta weights from predefined regions.

Our analyses primarily focused on predefined regions; however, to examine what other regions might be involved, we also conducted exploratory analyses. Condition effects at each voxel were estimated according to the general linear model and regionally specific effects were compared using linear contrasts. Each contrast produced a statistical parametric map of the *t* statistic, which was subsequently transformed to a unit normal *Z*‐distribution. The contrast images were then used in a random effect analysis to determine which regions were the most consistently activated across subjects.

## Results and Discussion

### Analysis overview

For the behavioral analyses, the dependent measures were accuracy on the cued‐recall tests and RTs to give the correct answer. The independent variable was the type of training that preceded the test (restudy, test, or restudy plus test). We conducted the behavioral analyses two different ways. One involved analyzing all trials regardless of prior test success. The other involved just including those trials that were used in the fMRI analyses where we only analyzed those trials that had a prior test success (see Rowland and DeLosh, 2015).

For fMRI analyses, the dependent measure is BOLD response on restudy and correct test trials and the independent variable was subsequent memory performance (correct and incorrect) and three conditions (STT‐T, SST‐T, and SSS‐T).

### Behavioral results

The third column of Table 1 presents mean recall accuracy of all trials in each phase and each condition. For Phase 3, accuracy was significantly better for trials following restudy (SST‐T condition) than for those following a test (STT‐T condition), *t*_19_ = 5.441, *P *<* *0.001, *d *=* *1.24. Phase 4 contrasts three learning conditions rather than two. Here too there was a significant effect of type of learning, *F*_2,38_ = 8.19, *P *=* *0.001, $\eta_{p}^{2}=0.30$. Accuracy was significantly higher following either one restudy and one test (SST‐T) or following two restudies (SSS‐T) as compared to trials that followed two tests (STT‐T) (*t*_19_ = 4.02, *P *=* *0.001, *d *=* *0.91; *t*_19_ = 3.10, *P *=* *0.006, *d *=* *0.68). These results replicate prior results that failed to find a testing effect advantage with short‐retention intervals (e.g., Wheeler et al. 2003). Here, we also failed to observe a testing advantage at a longer interval (24 h). This pattern can be understood as resulting from poor learning during the initial study phase combined with no feedback after testing (Rowland and DeLosh, 2014).

**Table 1:** Mean accuracy of all trials and of trials following correct test(s), mean correct RTs, and mean number of items correctly recalled for each phase of experiment.^a^

Phase	Condition	All trials	Trials following correct test(s)	Mean RTs for correct trials (sec)	Number of correct trials^b^
Phase 2	STT‐T	0.59 (0.05)		4.77 (0.14)	40
Phase 3	STT‐T	0.45 (0.05)	0.76 (0.04)	4.11 (0.21)	31
	SST‐T	0.58 (0.05)		4.52 (0.17)	26
Phase 4	STT‐T	0.36 (0.06)	0.80 (0.03)	4.09 (0.12)	24
	SST‐T	0.43 (0.06)	0.74 (0.04)	4.01 (0.16)	19
	SSS‐T	0.54 (0.06)		4.45 (0.19)	13

RTs, reaction time.

a Standard errors are shown in parentheses.

b Number of correct trials at Phase 2 and Phase 3 are also the number of trials included in fMRI analyses. Note that the number of trials per condition is not a pure measure of accuracy because the number of trials was not balanced initially (see the Materials section).

The fourth column of Table 1 presents mean accuracy of trials following correct tests. Recall accuracy in Phase 3 was significantly better for trials that followed a previous correctly recalled test (STT‐T condition) than for trials that followed a restudy (SST‐T condition), *t*_19_ = 5.48, *P *<* *0.001, *d *=* *1.42. Recall accuracy in Phase 4 (final assessment on Day 2) also showed a significant effect of type of learning condition, *F*_2,38_ = 27.88, *P *<* *0.001, $\eta_{p}^{2}=0.60$, such that performance was better for items that had been recalled correctly at least once (STT‐T or SST‐T condition) compared with items that had only been restudied (SSS‐T condition), *t*_19_ = 5.77, *P *<* *0.001, *d *=* *1.47; *t*_19_ = 6.07, *P *<* *0.001, *d *=* *1.75. The difference between subsequent accuracy at Phase 4 when prior learning opportunities involved two tests (STT‐T condition) compared with when prior learning involved one restudy and one test (SST‐T condition) was not reliable, *P *=* *0.07. These results are consistent with (Rowland and DeLosh, 2014), in that when the analyses are conditionalized on initial retrieval success, there is a clear testing effect for both short and long intervals. However, as Rowland and DeLosh acknowledge, there are problems with each method of studying the testing effect.

The fifth column of Table 1 shows the RTs for correct test trials at each phase in each condition. RTs for the second test in the STT‐T condition were significantly faster than RTs for the first test in the SST‐T condition, *t*_19_ = 2.21, *P *=* *0.039, *d *=* *0.60. As for RTs in Phase 4, there was a significant effect of type of learning condition, *F*_2,38_ = 11.54, *P* < 0.001, $\eta_{p}^{2}=0.38$, such that RTs were faster for items that had been recalled correctly at least once (STT‐T or SST‐T condition) compared with items that had only been restudied (SSS‐T condition), *t*_19_ = 3.38, *P *=* *0.003, *d *=* *0.95; *t*_19_ = 4.26, *P *<* *0.001, *d *=* *1.01. There was not a reliable difference in correct RTs in Phase 4 when comparing prior learning opportunities that involved two tests (STT‐T condition) versus prior learning that involved one restudy and one test (SST‐T condition), *P *=* *0.33.

In the current study, all 34 answers from a list were displayed on the two sides of the computer screen during the recall task. Conceivably, subjects might attempt to recognize the answer rather than recall the answer to the test probe. This seems unlikely because there were too many items on both sides of the screen to inspect to make that a viable strategy and the RTs were too fast to support that conjecture either. The average RTs of correct test trials in the scanner (4.47 sec) was very close to the average RTs of correct test trials in Phase 4 (4.18 sec) in which subjects were asked to recall and type the whole target words without alternatives presented. In addition, only 3% of the trials were not responded within 8 sec. We also examined the RTs and accuracy for trials in the scanner with a target word on the left side of the screen (4.53 sec, 56%) and those with a target word on the right side of the screen (4.41 sec, 53%). We found no significant differences in either RTs or accuracy.

### Predefined fMRI analyses

#### Subsequent memory effects based on initial study and restudy trials

Although the primary focus of this investigation concerns the activation patterns during retrieval, for completeness we also analyzed the encoding trials (initial study trial and the restudy trials) as a function of subsequent memory performance. Specifically, we back sorted the encoding trials into those that were successfully recalled in the subsequent phase and those that were not correctly recalled, comparing the brain activity during encoding for these two groups of trials. For the initial study phase (Phase 1), we obtained significant subsequent memory effects in left PFC (*t*_18_ = 5.28, *P *<* *0.001, *d *=* *1.21), left PPC (*t*_18_ = 2.23, *P *=* *0.039, *d *=* *0.51), and left HPC (*t*_18_ = 2.58, *P *=* *0.019, *d *=* *0.59). No significant subsequent memory effects were found in any other regions.

Unlike the initial study trials, no significant subsequent memory effects were found for any ROIs in the restudy conditions. Conceivably, after an initial study subjects have the illusion that they have already learned the information adequately. That is, unless subjects are tested on the material, they do not appreciate that restudying will help them better learn the material. This explanation fits with previous studies on metacognition that suggest that subjects have an “illusion of knowing” (Glenberg et al. 1982).

#### Subsequent memory effects based on activity patterns during test for well‐learned versus less well‐learned answers

Our main focus is concerned with the neural patterns during testing. In previous research we have shown that the activation pattern during retrieval can predict whether the next recall is correct (Liu et al. 2014), but this was done for items that were not overlearned. In the current study, we investigated whether the activation pattern during a correct recall will continue to predict whether the next subsequent recall will be correct when the recall process is too easy, that is, subjects sense that the information is well learned. It might seem odd to ask whether recall activation can still predict subsequent accuracy when the answer is well learned. One might suppose that since the information is so well learned that performance has “hit ceiling” and there will not be any variability to account for. In fact, we found that we could reliably predict whether later retrievals would be correct based on activation patterns during current retrieval and based on the strength of the current memory trace.

We operationalized the strength of the correct answer based on whether it had previously been correctly recalled once or twice. That is, we contrasted the activation patterns during retrieval for items that were then correctly recalled for the first time with items that had already been correctly recalled once before.

This contrast involved two different learning conditions of word pairs during Phase 3 (see Fig. 1, top two rows). For both conditions, trials that produced correctly recalled word pairs were back sorted as a function of whether or not they were correctly recalled again on the final assessment (Phase 4, Day 2). These subsequent memory contrasts were examined in six predefined ROIs discussed previously.

**Figure 2:**
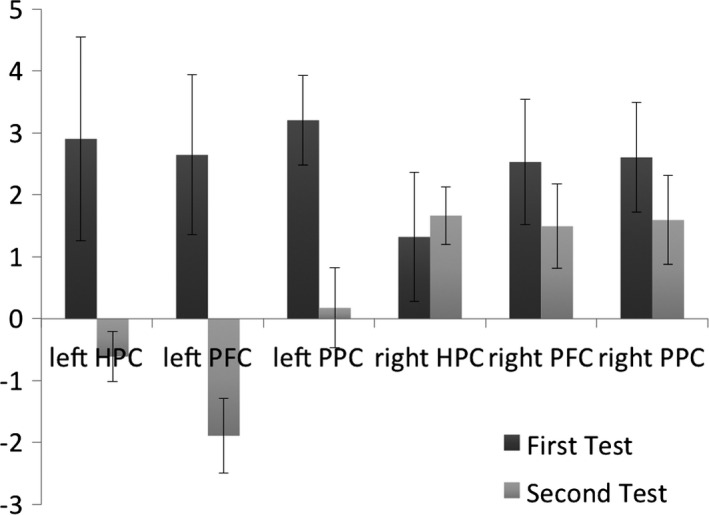
Subsequent memory effect (beta values of ROIs for subsequently correct trials minus these for subsequently incorrect trials) for the first test in the SST‐T condition versus the second test in the STT‐T condition. For the first test, significant or marginally significant subsequent memory effects were observed in all six ROIs (left PFC,* t*_18_ = 4.69, *P *<* *0.001, *d *=* *1.08; right PFC,* t*_18_ = 2.64, *P *=* *0.017, *d *=* *0.61; left PPC,* t*_18_ = 2.85, *P *=* *0.011, *d *=* *0.67; right PPC,* t*_18_ = 3.12, *P *=* *0.006, *d *=* *0.72; left HPC,* t*_18_ = 1.87, *P *=* *0.078, *d *=* *0.43; right HPC,* t*_18_ = 2.17, *P *=* *0.044, *d *=* *0.50). For the second test, significant subsequent memory effects were observed in right HPC (*t*_18_ = 2.92, *P *=* *0.009, *d *=* *0.66), right PFC (*t*_18_ = 2.33, *P *=* *0.032, *d *=* *0.54), and right PPC (*t*_18_ = 2.36, *P *=* *0.03, *d *=* *0.54). The left PFC showed a subsequent failure effect (*t*_18_ = 3.11, *P *=* *0.006, *d *=* *0.71). HPC, hippocampus; PFC, prefrontal cortex; PPC, posterior parietal cortex; ROI, regions of interest.

Figure 2 plots the differences in activation during the Phase 3 test between trials for which the answer was again correct at Phase 4 (Day 2) and those trials that switched from correct at Phase 3 to incorrect at Phase 4 in the SST‐T condition (dark bars) and the STT‐T condition (light bars), respectively. When the difference was greater than zero, we defined this as a subsequent memory effect and when the difference was less than zero, we defined this as a subsequent failure effect. For activation during Phase 3 in the SST‐T condition, significant or marginally significant subsequent memory effects were observed in all six ROIs. In the STT‐T condition of Phase 3, significant subsequent memory effects were observed in right HPC, right PFC, and right PPC. The left PFC showed a subsequent failure effect. No significant effects were found in left PPC or left HPC. The inferential statistics are reported in Figure 2.

The results of the first test were consistent with prior results on the neural mechanisms underlying the testing effect (van den Broek et al. 2013; Wing et al. 2013; Liu et al. 2014) that the brain regions previously identified as responsible for learning during study (Fletcher et al. 1998; Blumenfeld and Ranganath 2007; Kim 2011; Manelis et al. 2013), namely left PFC, left PPC, and HPC are also involved during the testing phase. Furthermore, we found that activation in right PFC and right PPC also predicts subsequent correct recall based on testing trials. This new paradigm also enabled us to examine the difference between the subsequent memory patterns in the first test and that in the second test. When examining subsequent memory effects on the second test, higher activation in the right hemisphere was still associated with correct recall on the final test; however, higher activation in left PFC was associated with subsequent failure.

#### Subsequent memory effects of the first test partitioned on subsequent recall after short versus long delay

Although not initially planned, our paradigm also allowed us to examine the effect of retention length between the initial tests and final assessment. Prior behavioral studies have found contradictory effects of testing on short‐term retention with some research finding benefits of testing only after a long lag between practice tests and final tests (e.g., Wheeler et al. 2003) and others finding a testing effect advantage over restudy even at short delays (Verkoeijen et al. 2012; Rowland and DeLosh 2015).

Given the nature of our design, it is possible to examine the neural activity during testing to determine whether the subsequent memory effects that predict subsequent correct recall differ in these two situations.

**Figure 3:**
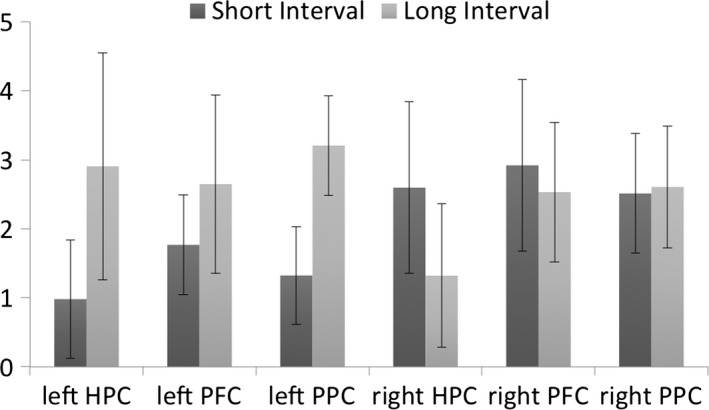
Subsequent memory effect (parameter estimates of regions of interests for subsequently correct trials minus these for subsequently incorrect trials) for recall at Phase 2 in the STT‐T condition (testing effect at short interval) and at Phase 3 in the SST‐T condition (testing effect at long interval). The subsequent memory effect was larger for the long‐delay condition compared to the short‐delay condition in left prefrontal cortex (*t*_18_ = 2.28, *P *=* *0.035, *d *=* *0.57).

We contrasted the subsequent memory effect patterns in the six predefined ROIs during successful recall at Phase 2 in the STT‐T condition (short‐term testing effect) partitioned on accuracy of Phase 3 and at Phase 3 in the SST‐T condition (long‐term testing effect) partitioned on accuracy of Phase 4 (shown in Fig. 3). In left PFC, the subsequent memory effect was larger for the long‐delay condition compared to the short‐delay condition (*t*_18_ = 2.28, *P *=* *0.035, *d *=* *0.53). Given that left PFC is primarily involved in encoding (Fletcher et al. 1998; Blumenfeld and Ranganath 2007; Kim 2011), the larger effect in left PFC for the longer delay condition may indicate that re‐encoding is more important for long‐term retention compared to short‐term retention.

There were no other significant between‐condition differences in any other ROIs. It is interesting to note that these fMRI results showed a similar subsequent memory effect pattern when partitioned on subsequent performance after either a short or long delay. These results support the results of Rowland and DeLosh (2014) who found that testing promotes both short‐term and long‐term memory retention.

### Exploratory fMRI analyses

**Figure 4:**
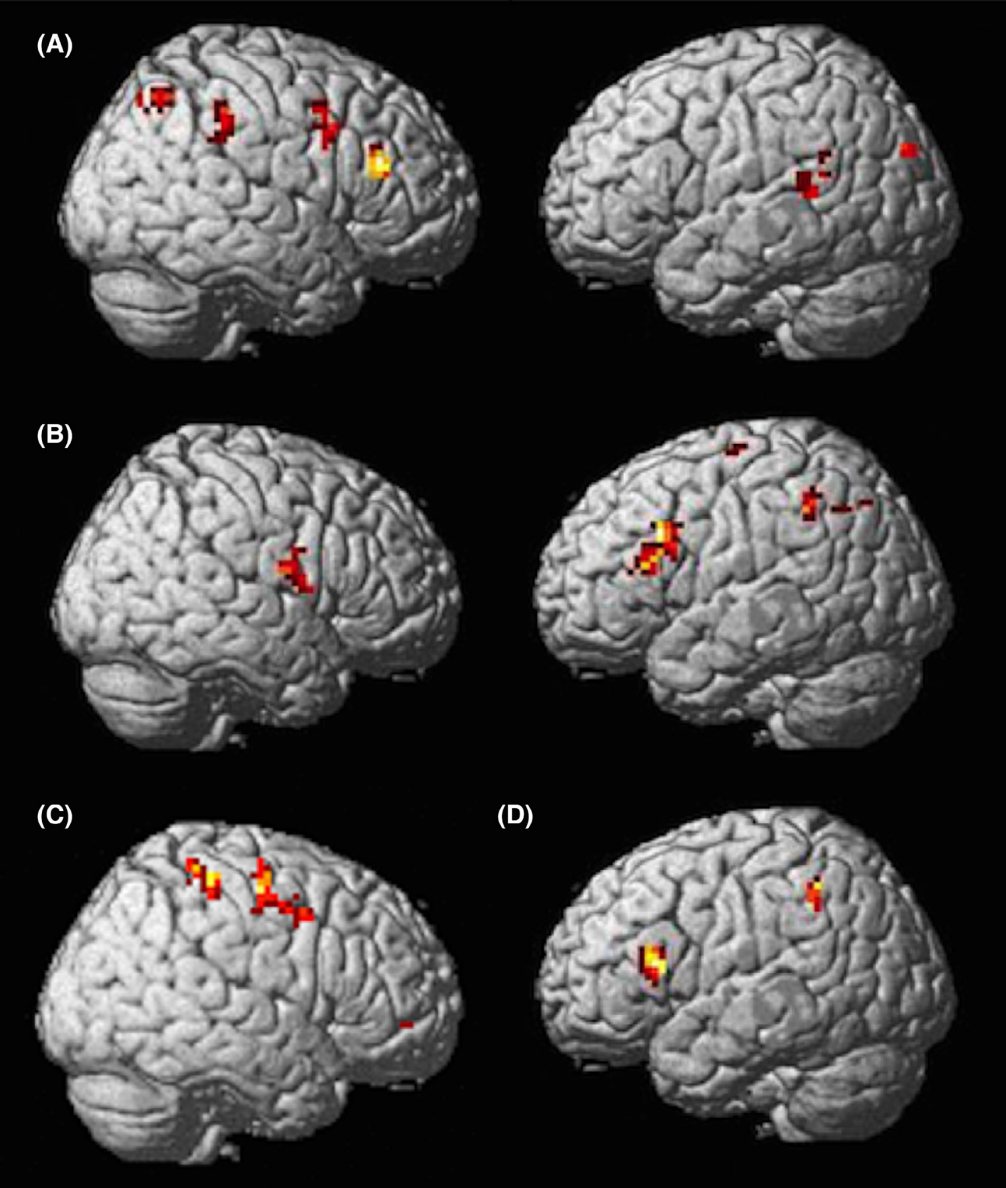
Subsequent memory/failure effects. (A) The subsequent memory effect of the first test in the STT‐T condition. (B) The subsequent memory effect of the first test in the SST‐T condition. (C) The subsequent memory effect of the second test in the STT‐T condition. (D) The subsequent failure effect of the second test in the STT‐T condition.

In order to insure that our planned analyses for specific regions did not obscure other possible findings, we also conducted exploratory, whole brain analyses in the same manner as those conducted for each of the contrasts used with predefined ROI analyses. An alpha level of *P* < 0.001 was used in this analysis. To correct for multiple comparisons, only those regions having a contiguous cluster size of 10 or more significant voxels are reported. This threshold yielded a corrected threshold of *P* < 0.05, determined by a Monte Carlo simulation using the AlphaSim program (Cox and Hyde, 1997). Table 2 and Figure 4 present the regions that showed significant effects for each of the contrasts using this criterion. We examined the subsequent memory effect in each test condition. As shown in Figure 4, all of the regions identified in this contrast show the same pattern that we found for the predefined regions, bilaterally, when analyzing the subsequent memory effects for the first test that was partitioned on either a short (Fig. 4A) or long delay (Fig. 4B). For the first test in the STT‐T condition, predefined ROIs, right PFC (BA 49, BA 9), bilaterally PPC (BA 40, BA 7, BA 19) were also identified in whole brain analysis. In addition to predefined regions, left temporal gyrus (BA 13, BA 22) and right parahippocampal gyrus (BA 27) also showed subsequent memory effect. For the first test in the SST‐T condition, predefined regions, left PFC (BA 9, BA 46), left PPC (BA 40, BA 19) were identified in whole brain analysis. In addition, bilateral precentral gyrus (BA 6) also showed subsequent memory effect. For the second test, a subsequent memory effect was only found in right hemisphere (BA 6, BA 10, BA 2) (Fig. 4C) and a subsequent failure effect was found in left PFC (BA 45) and left PPC (BA 40) (Fig. 4D).

**Table 2:** Regions showing subsequent memory/failure effects in each testing condition in the exploratory analyses

Regions	L/R	BA	MNI coordinates			*T* scores	Cluster
Subsequent memory effect of initial study							
Inferior temporal gyrus	L	37	−48	−60	−15	6.77	14
	L	20	−54	−51	−15	5.31	
Inferior frontal gyrus	L	46	−51	27	21	5.92	10
Precentral gyrus	L	44	−45	6	9	5.20	16
	L	6	−51	−3	9	3.69	
Subsequent memory effect of the first test in the STT‐T condition							
Superior temporal gyrus	L	13	−42	−48	24	6.32	10
		13	−48	−48	18	5.88	
Superior temporal gyrus	L	22	−66	−42	18	6.26	12
Cuneus	L	19	−30	−87	30	6.1	15
		18	−21	−81	24	5.78	
Middle frontal gyrus	R	46	51	30	21	6.6	48
Middle frontal gyrus	R	6	39	6	42	5.88	30
		9	45	9	36	5.72	
Inferior parietal lobule	R	40	45	−36	39	6.48	47
		40	39	−33	33	5.98	
		40	42	−42	30	5.38	
Precuneus	R	7	21	−66	51	6.08	17
Superior parietal lobule	R	7	33	−63	51	5.8	22
Parahippocampal gyrus	R	27	21	−30	−6	6.26	11
Subsequent memory effect of the first test in the SST‐T condition							
Middle frontal gyrus	L	9	−54	18	33	9.82	72
		9	−45	15	33	8.55	
		46	−42	24	18	7.45	
Precentral gyrus	L	6	−18	−18	66	9.67	27
Precuneus	L	19	−30	−63	39	7.89	29
Inferior parietal lobule	L	40	−33	−45	45	6.73	42
		40	−48	−45	42	6.52	
Precentral gyrus	R	6	51	−3	18	7.05	39
		6	42	−6	27	6.86	
		44	51	3	12	6.29	
Subsequent memory effect of the second test in the STT‐T condition							
Medial frontal gyrus	R	6	3	14	46	6.70	18
Middle frontal gyrus	R	6	24	−1	44	5.59	24
		6	36	−1	39	5.79	
		6	33	5	47	5.13	
Middle frontal gyrus	R	6	33	−9	47	6.82	53
		6	53	2	41	6.22	
		4	48	−6	47	5.88	
Medial frontal gyrus	R	6	15	3	52	6.49	17
Superior frontal gyrus	R	10	21	47	−2	6.19	10
Postcentral gyrus	R	2	36	−35	60	7.06	34
		3	42	−26	57	5.98	
Subsequent failure effect of the second test in the STT‐T condition							
Inferior frontal gyrus	L	45	−48	21	15	9.96	50
		45	−54	21	6	7.24	
Inferior parietal lobule	L	40	−45	−45	42	7.52	21
		40	−54	−48	51	6.14	

MNI, Montreal Neurological Institute.

## General Discussion

In this experiment, we wanted to explore the contribution of both the retrieval and re‐encoding processes during test as well as the restudy processes on subsequent learning of the material. Overall, we found that activity of more brain regions during test trials predicts subsequent memory performance than brain activity during restudy trials. In addition, we found that brain regions in both the left and right hemispheres showed more activation during the first correct retrieval for test trials that were again remembered on a later test as compared to correct retrievals that were subsequently forgotten on that later test.

On the other hand, after subjects had already correctly recalled the answer in Phase 2, the activation pattern during testing in Phase 3 for correct answers (the second test in the STT‐T condition) showed a different pattern: While regions in the right hemisphere still showed a positive relationship between activation and likelihood of recalling it correctly for the third time, regions in the left hemisphere, especially left PFC, showed the inverse pattern.

How does one explain this dissociative pattern? One possibility is that there are two processes underlying learning from tests. One mechanism would involve the *unique process of retrieval* as proposed by the elaborative retrieval account (Anderson and Reder 1979; Carpenter and DeLosh 2006; Carpenter 2009). Given that the subsequent memory effect in the right hemisphere was only observed during testing, we propose that the retrieval process is reflected in right PFC and other regions in the right hemisphere. The other mechanism would involve *the re‐encoding of the information after successful retrieval* as proposed by the reconsolidation account (Finn and Roediger 2011). In the current study, the re‐encoding process is reflected in left PFC, which has commonly been associated with memory encoding (Fletcher et al. 1998; Blumenfeld and Ranganath 2007; Kim 2011).

An important qualification that we wish to highlight is that this re‐encoding process might not always engage in a productive way especially when the answer seems overlearned to the person retrieving the information. While we are suggesting that the learner may not be motivated to re‐encode the information when well learned, we are not postulating that this behavior is conscious. This tacit boredom response may well be automatic. To test this hypothesis, we operationalized overlearning to refer to those trials for which the answer had already been recalled correctly two times. Whether conscious or not, subjects might be less motivated to continue restudying those pairs, resulting in their minds wandering to other things. If so, activation in the regions associated with encoding would represent thinking about other things.

Part of the test of this hypothesis was to back sort just those trials that had already been correctly recalled twice, and to examine the activation patterns after retrieval as a function of whether the third recall was subsequently answered correctly or wrong. Here, we found the opposite pattern that we had observed after the first successful recall: More activation in left PFC following a second correct test was associated with a lower probability of getting the answer correct on the third test. We call this the *subsequent failure effect*.

To summarize, we interpret this greater activation in the left hemisphere after two correct retrievals as support for the idea that encoding irrelevant information produces interference later. It is unclear whether diminishing returns of additional tests results from mind‐wandering postretrieval or whether the mind does not engage in further efforts because it tacitly knows that additional restudy has less benefit. Regardless, we suspect that when the mind wanders as opposed to refocusing on studying the relevant information, the benefits from additional tests may be compromised by the interference introduced from thinking of other things in that context.

This explanation is supported by the behavioral results that neither RTs nor accuracy were reliably different for tests at Phase 4 that followed two tests (STT‐T condition) compared with those that followed one test (SST‐T), suggesting that the benefit of the second test was compromised by encoding irrelevant information. The interference explanation is also consistent with the literature. Prior behavioral research (e.g., Roediger and Marsh 2005) showed that multiple choice testing might create false memories when the number of lures is large. In addition, prior animal research (Karlsson and Frank 2009) also showed that during retrieval, irrelevant environmental information could be associated with remembered information.

Our two‐process explanation is consistent with the HERA (hemispheric encoding/retrieval asymmetry) model of Tulving et al. (1994). That model posited that left PFC is preferentially involved in the encoding of new information into episodic memory and right PFC is more involved in episodic memory retrieval. Our results suggest that activity in right PFC reflects the retrieval attempt process and the left PFC reflects the postretrieval re‐encoding process. However, more direct evidence is still needed to support this explanation for the subsequent failure effect.

In conclusion, our results indicate that left PFC activation during testing predicts subsequent success after the first correct test, but not after multiple correct answers. In fact, greater activation in this region after several correct recalls predicted subsequent failure. Our interpretation is that, with overlearning, subjects are unmotivated to restudy and the activation that is observed is indicative of mind‐wandering to other things that can lead to interference.

It is important to note that, unlike the left PFC, activation in regions in the right hemisphere consistently predict subsequent success regardless of whether it was the first or second correct recall. Based on this pattern, we propose that there are two distinct processes underlying the testing effect, namely a retrieval process that brings the answer to mind and a re‐encoding process for additional study after retrieval. Subjects will always benefit from a successful retrieval process given that it necessarily strengthened the correct retrieval route to the answer.

## Conflict of Interest

None declared.

## Note

1 We did not equate the procedures for test and restudy trials because we did not directly compare brain activity between test and restudy trials.
